# Komodo dragon-inspired synthetic peptide DRGN-1 promotes wound-healing of a mixed-biofilm infected wound

**DOI:** 10.1038/s41522-017-0017-2

**Published:** 2017-04-11

**Authors:** Ezra M. C. Chung, Scott N. Dean, Crystal N. Propst, Barney M. Bishop, Monique L. van Hoek

**Affiliations:** 1 College of Science, George Mason University, Manassas, VA 20110 USA; 2 School of Systems Biology, George Mason University, Manassas, VA 20110 USA; 3 Department of Chemistry and Biochemistry, George Mason University, Manassas, VA 20110 USA; 4 Present Address: Director of Research and Development, STCube Pharmaceuticals, Inc., 401 Professional Dr. Suite 108, Gaithersburg, MD 20879-3429 USA

**Keywords:** Biofilms, Antimicrobials, Pathogens, Applied microbiology

## Abstract

Cationic antimicrobial peptides are multifunctional molecules that have a high potential as therapeutic agents. We have identified a histone H1-derived peptide from the Komodo dragon (*Varanus komodoensis)*, called VK25. Using this peptide as inspiration, we designed a synthetic peptide called DRGN-1. We evaluated the antimicrobial and anti-biofilm activity of both peptides against *Pseudomonas aeruginosa* and *Staphylococcus aureus*. DRGN-1, more than VK25, exhibited potent antimicrobial and anti-biofilm activity, and permeabilized bacterial membranes. Wound healing was significantly enhanced by DRGN-1 in both uninfected and mixed biofilm (*Pseudomonas aeruginosa* and *Staphylococcus aureus*)-infected murine wounds. In a scratch wound closure assay used to elucidate the wound healing mechanism, the peptide promoted the migration of HEKa keratinocyte cells, which was inhibited by mitomycin C (proliferation inhibitor) and AG1478 (epidermal growth factor receptor inhibitor). DRGN-1 also activated the EGFR-STAT1/3 pathway. Thus, DRGN-1 is a candidate for use as a topical wound treatment. Wound infections are a major concern; made increasingly complicated by the emerging, rapid spread of bacterial resistance. The novel synthetic peptide DRGN-1 (inspired by a peptide identified from Komodo dragon) exhibits pathogen-directed and host-directed activities in promoting the clearance and healing of polymicrobial (*Pseudomonas aeruginosa* & *Staphylococcus aureus*) biofilm infected wounds. The effectiveness of this peptide cannot be attributed solely to its ability to act upon the bacteria and disrupt the biofilm, but also reflects the peptide’s ability to promsote keratinocyte migration. When applied in a murine model, infected wounds treated with DRGN-1 healed significantly faster than did untreated wounds, or wounds treated with other peptides. The host-directed mechanism of action was determined to be via the EGFR-STAT1/3 pathway. The pathogen-directed mechanism of action was determined to be via anti-biofilm activity and antibacterial activity through membrane permeabilization. This novel peptide may have potential as a future therapeutic for treating infected wounds.

## Wound therapy: Healing from a Komodo dragon

A synthetic peptide based on a natural molecule found in the Komodo dragon promotes healing of biofilm-infected wounds. Peptides are small protein-like molecules. Monique van Hoek, Barney Bishop and colleagues at George Mason University in Virginia, USA, isolated a natural peptide with some antimicrobial properties from Komodo dragon plasma. They designed a modified synthetic version with rearranged amino acids, named DRGN-1 in recognition of the “Komodo dragon” peptide that inspired it. In preliminary trials, DRGN-1 enhanced the healing of biofilm-infected wounds in mice, and was more effective than the natural peptide. This may be due to both bacterial- and host-directed effects. DRGN-1 reduced biofilm and bacterial number while increasing wound closure. The authors suggest DRGN-1 could be developed into a therapeutic agent that may treat the biofilm-infected wounds that are increasingly resistant to conventional antibiotics.

## Introduction

The increasing prevalence of multidrug-resistant (MDR) pathogens demands new antibiotics. Due to their potent and broad antimicrobial activity, antimicrobial peptides (AMPs), and in particular cationic antimicrobial peptides (CAMPs), are a possible alternative to conventional antibiotics. Although the mode of action of AMPs is not fully understood, it is believed that their major targets are the cytoplasmic membrane and intracellular molecules.^1^ In addition, AMPs can have both antimicrobial and antibiofilm activities, and that antimicrobial and antibiofilm activity may be independent mechanisms of the same peptide.^1–5^ It is also believed that it is difficult for bacteria to develop resistance against CAMPs that kill quickly through their actions on the cytoplasmic membrane or that act through complex mechanisms inside the bacterial cell.^5^ Antimicrobial peptides have been found to have a broad-spectrum activity, with active peptides identified against both gram-positive and gram-negative bacteria, membrane-bound viruses, and fungi. Antimicrobial peptides can also have significant immunomodulatory activity directed towards the host, which is likely to be an additional critical aspect of their function in vivo.^6–8^ Some peptides have been identified as having a role in promoting wound-healing.^9^ For these reasons, AMPs are potentially useful in combating MDR bacteria and may be useful as potential treatments for infected wounds.

Animals are a promising source of CAMPs because of their evolved defenses to bacteria. The American alligator and other crocodilians are evolutionarily ancient animals, whose plasma and leukocyte extracts have been previously shown to have antimicrobial activity.^10–12^ Discovering and identifying CAMPs from blood, however, is labor-intensive and expensive. We developed a new bioprospecting-particle and proteomics approach to CAMP discovery.^13^ This platform includes the ability to harvest efficiently from small sample volumes (e.g., 100 µl), advanced middle-down mass spectrometry techniques, and the identification of CAMPs through de novo peptide sequencing.

In the present work, we applied this process to the discovery of novel AMPs from plasma obtained from another reptile, the Komodo dragon (*Varanus komodoensis*), a large species of lizard found on the Indonesian island of Komodo. The saliva of wild Komodo dragons is thought to contain many different strains of bacteria, some of which are known to cause sepsis.^14, 15^ However, this bacteria-laden saliva never sickens the Komodo dragon. Investigators therefore hypothesized that proteins in the dragon's saliva or blood might provide immunity. Using our AMP discovery bioprospector platform,^13^ we identified a Komodo dragon peptide from Komodo plasma that we named VK25 (Fig. S1). Inspired by this VK25 peptide, we designed and created a new short synthetic peptide, which is a rearrangement of the two amino acids at the N-terminal of the VK25 peptide, and which we named DRGN-1. DRGN-1 exhibited promising antimicrobial and anti-biofilm properties. Moreover, the DRGN-1 peptide significantly promoted wound healing in vitro and in vivo, in both uninfected and mixed biofilm infected wounds.

## Results

### Antimicrobial activity of DRGN-1

To investigate whether DRGN-1 could exert antimicrobial effects, we tested the activity of CAMPs against the gram-negative *Pseudomonas aeruginosa*, *Francisella novicida* and *Burkholderia thailandensis* and the gram-positive *Staphylococcus aureus*. It should be noted that VK25 is a histone H1-derived peptide from Komodo, and histone peptides are generally weakly active or inactive under minimal inhibitory concentration (MIC) conditions, but may still have activity under low-salt, phosphate buffer conditions (EC_50_).^16, 17^ The results showed that DRGN-1 demonstrated the antimicrobial activity in an EC_50_ assay against the gram-negative bacterium *P. aeruginosa* (but not *F. novicida* or *B. thailandensis*) as well as the gram-positive bacterium *S. aureus* within the range of 0.77–7.1 µg/ml (0.50–4.62 µM). This was compared to VK25, which was significantly less active with anti-*Pseudomonas* activity in the range of 25–30 µg/ml and anti-*Staphylococcus* activity of more than 100 µg/ml (Table 1). These peptides do not show antimicrobial activity in full broth under MIC conditions, as is known for many other peptides, including the human cathelicidin LL-37.^17, 18^

**Table 1:** Antimicrobial (EC_50_) activity of DRGN-1, VK25 and LL-37 against a panel of gram-positive and gram-negative bacteria

Species	DRGN-1			VK25			LL-37		
	EC_50_ (µM)	EC_50_ (µg/ml)	95% CI (µg/ml)	EC_50_ (µM)	EC_50_ (µg/ml)	95% CI (µg/ml)	EC_50_ (µM)	EC_50_ (µg/ml)	95% CI (µg/ml)
*P. aeruginosa* PAO1	4.46	6.85	4.22–11.1	17.7	27.2	6.86–108	ND	ND	–
*P. aeruginosa* ATCC 9027	2.86	4.39	1.12–17.1	17.2	26.4	14.1–49.4	0.525^a^	2.36^a^	2.00–2.76
*P. aeruginosa* PAO1 (pTDK-GFP)	4.62	7.10	3.46–14.6	20.0	30.8	11.2–84.5	0.599	2.69	1.32–5.50
*F. novicida* U112	>65	>100	–	>65	>100	–	0.0534^b^	0.24^b^	0.18–0.30
*B. thailandensis E264*	>65	>100	–	>65	>100	–	1.88	8.43^c^	5.37–13.2
*S. aureus* ATCC 25923	2.63	4.04	(wide)	>65	>100	–	0.552^a^	1.27^a^	0.44–3.70
*S. aureus* SH1000 (pAH9-RFP)	0.50	0.77	0.25–2.4	>65	>100	–	ND	ND	–

This table indicates the EC_50_ and 95% confidence interval (CI) of the peptides (in µg/ml) against bacteria in an antimicrobial assay, and also in µM, *n* = 3

Previously determined by us: ^a^ from ref. 13, ^b^ from ref. 2, ^c^ from ref. 69

*ND* not determined

### Anti-biofilm activity of DRGN-1

**Fig. 1:**
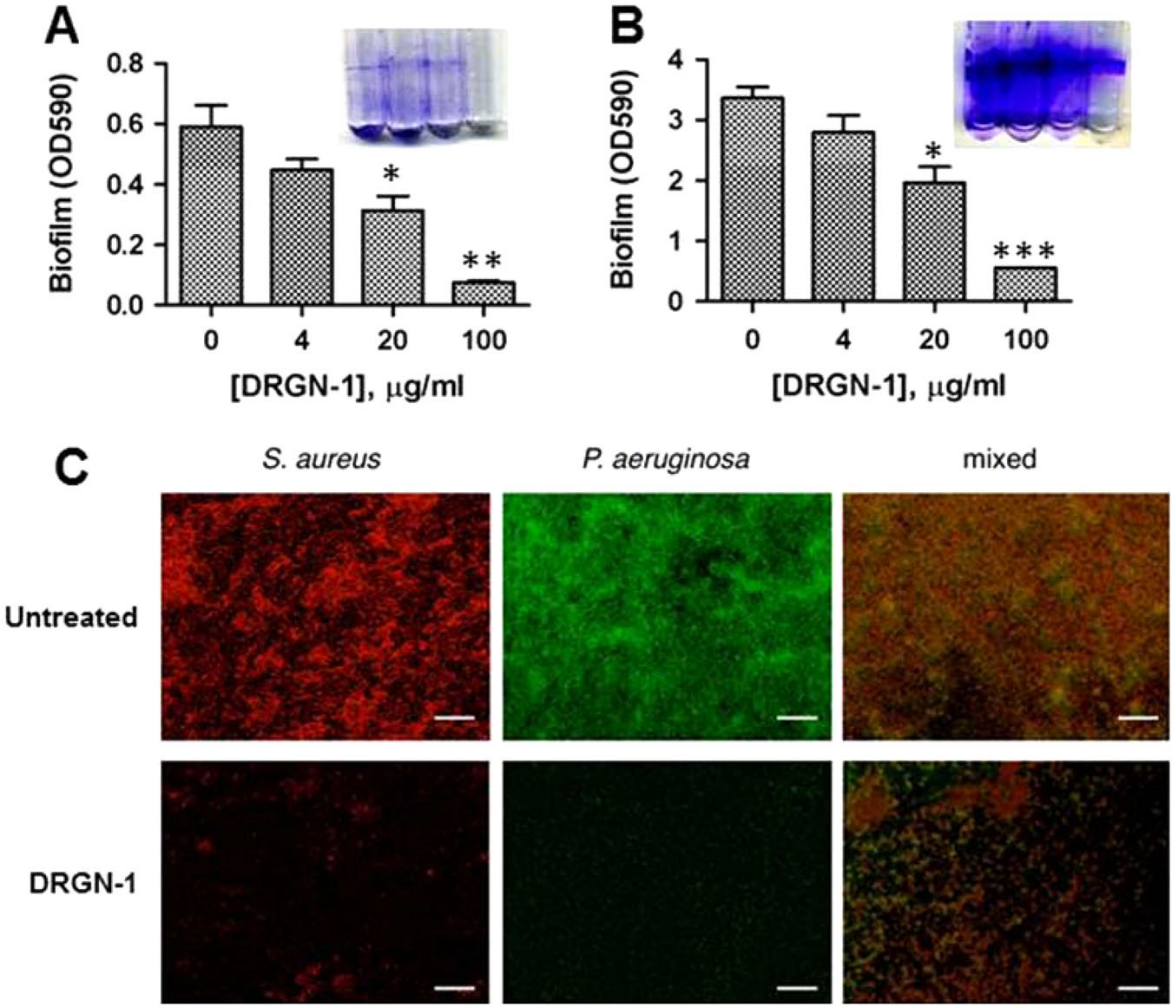
Anti-biofilm activity of DRGN-1. **a** DRGN-1 anti-biofilm activity against *S. aureus* using three interdependent methods. The activity was analyzed by crystal violet staining of biofilm in culture tube at 24 h. **b** DRGN-1 anti-biofilm activity against *P. aeruginosa*. Pictures (*right inlet* in **a** and **b**) are a representative biofilm stained with crystal violet. *P* values were calculated using a two-tailed *t* test (assuming unequal variances) comparing test strains to untreated control (*P < 0.05; **P < 0.01; ***P < 0.001). **c** Biofilm evaluation by confocal microscopy. Representative images of biofilms of *P. aeruginosa* PAO1-pTDK-GFP expressing GFP, *S. aureus* SH1000 pAH9-RFP expressing RFP or mixed cultures exposed to PBS (control) or DRGN-1 (25 µg/ml) for 24 h. DRGN-1-exposed biofilms are thinner and more sparse than untreated controls. Scale bar: 20 µm

Next, the ability of DRGN-1 to prevent the formation of biofilm after 18 h was examined by crystal violet staining and confocal microscopy (Fig. 1). When cultured in a polystyrene tube under shaking conditions, *P. aeruginosa* and *S. aureus* form biofilms along the sidewall of the culture tube, which was strongly inhibited by DRGN-1 in a concentration-dependent manner (Fig. 1a, b). For confocal imaging, DRGN-1 (final concentration, 25 μg/ml) was incubated with bacteria overnight in the wells of a chamber slide. *P. aeruginosa* Green-fluorescent protein (GFP)^+^ and *S. aureus* Red-fluorescent protein (RFP)^+^ were used for these studies.^19^ The cells were incubated for 24 h in tryptic soy broth (TSB) medium in the presence of DRGN-1. The next day, the wells were rinsed and the development of a biofilm was compared with a control well (without DRGN-1). Biofilms of both *P. aeruginosa* and *S. aureus* were significantly reduced by DRGN-1. In mixed-culture biofilms grown for 24 h without treatment, *P. aeruginosa* significantly outgrows *S. aureus*,^19^ thus the appropriate starting ratio was established as a 99:1 ratio *S. aureus* to *P. aeruginosa*. Treatment with DRGN-1 had a significant inhibitory effect on the mixed bacterial cultures in contrast with untreated control.

### Ability of DRGN-1 to affect *P. aeruginosa*-infected HEKa keratinocytes

**Fig. 2:**
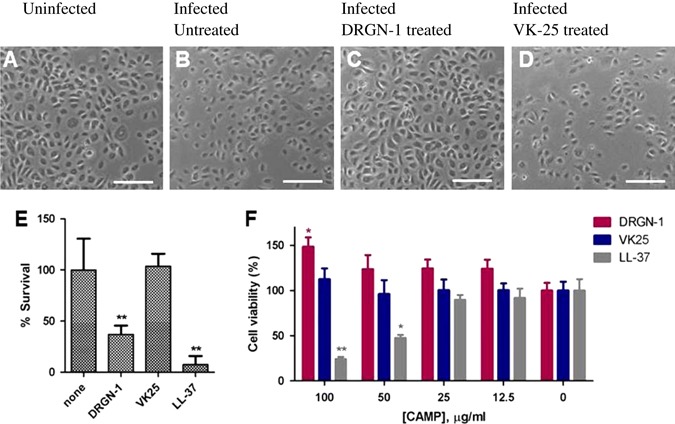
Treatment of cells with peptide reduces intracellular *P. aeruginosa* bacteria. **a** Light microscopy images of uninfected HEKa keratinocytes. **b**–**d** About 100,000 cells were infected with *P. aeruginosa* ATCC 9027 (1 × 10^7^ CFU/ml) for 2 h, washed with PBS, treated with gentamicin to remove extracellular bacteria for 1 h, and **b** not-treated, or **c** treated with DRGN-1 (30 µg/ml), or (D) VK25 (30 µg/ml) for 2 h at 37 °C. **e** Bacterial survival within infected HEKa keratinocytes upon peptide treatment at 2 h post treatment. 100% represents the number of bacteria found inside of untreated HEKa cells. **f** Cytotoxicity testing: the peptides were tested for their cytotoxic effect against HEKa cells. The number of metabolically active cells remaining was determined by the MTT assay and is expressed as percentage with respect to the non-peptide-treated control cells. *P* values were calculated using a two-tailed *t* test (assuming unequal variances) comparing test strains to untreated control (*P < 0.05; **P < 0.01)

To gain insight into the ability of DRGN-1 to exert activity against internalized *P. aeruginosa*, HEKa keratinocytes were infected by *P. aeruginosa* ATCC 9027, and subsequently treated with DRGN-1 or VK25. As highlighted in Fig. 2, the killing of infected keratinocytes was pronounced 2 h after their exposure to the bacteria, presumably due to *P. aeruginosa*-induced apoptotic cell death, which was strongly prevented by exogenous treatment with DRGN-1 (Fig. 2a–d). Exogenous application of DRGN-1, but not VK25, significantly reduced the number of internalized bacteria with the peptide causing 64% reduction in survival of the internalized bacteria at the concentration used (30 µg/ml) after 2 h (Fig. 2e). LL-37 treatment of infected HEKa cells also caused a significant reduction in internalized *P. aeruginosa* cells. Both of these reductions were statistically significant compared to untreated or VK-25-treated cells. We conclude that DRGN-1 treatment of *P. aeruginosa*-infected HEKa keratinocytes leads to increased killing of those bacteria, whether directly by the peptide (direct antibacterial effect) or by a peptide-induced response of the keratinocyte cells (host-directed effect).

The cytotoxic effect of DRGN-1 and VK25 on HEKa cells was studied using an MTT (3-(4, 5-dimethylthiazolyl-2)- 2,5-diphenyltetrazolium bromide) based assay. As shown in Fig. 2f, both DRGN-1 and VK25 were devoid of toxic effects against these mammalian cells at a concentration range of 12.5–100 µg/ml. However, the control CAMP, LL-37, caused a significant decrease in HEKa cell viability at concentrations ranging from 50–100 µg/ml (Fig. 2f). DRGN-1 also did not show any toxicity against erythrocytes in a hemolysis assay at a high concentration (Fig. S2).

### Effects of DRGN-1 on membrane permeability

**Fig. 3:**
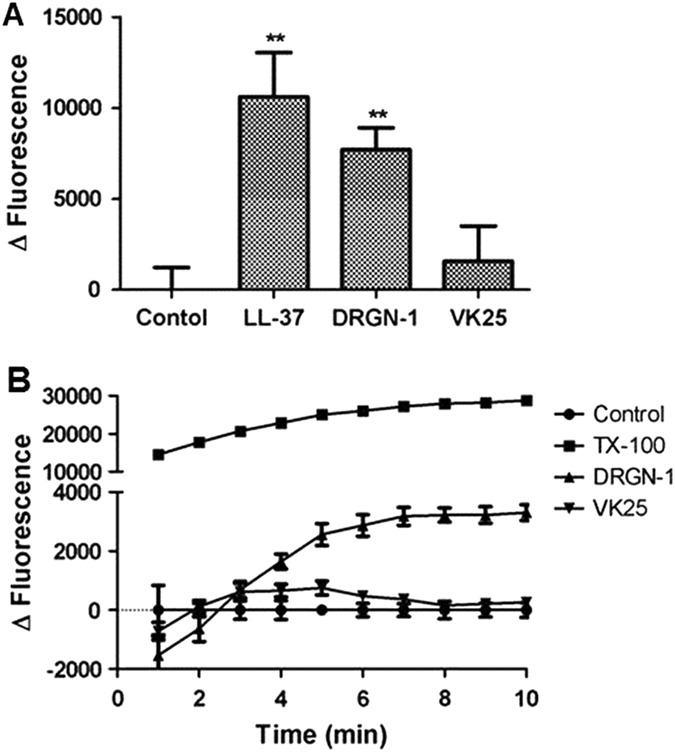
Membrane permeability, and membrane potential. **a** Fluorescent dye uptake for EtBr by the *P. aeruginosa* ATCC 9027 in the presence of peptides (20 µg/ml). *P* values were calculated using a two-tailed *t* test (assuming unequal variances) comparing with untreated control at 20 min (**P < 0.01). **b** Membrane depolarization was monitored by the change in the intensity of fluorescence emission of the membrane-potential-sensitive dye DiSC_3_(5) (excitation wavelength at 622 nm, emission wavelength at 670 nm) on addition of 20 µg/ml peptides or 0.1% Triton X-100 for a positive control

Measuring the uptake of the fluorescent probe, ethidium bromide (EtBr), allowed us to assess the permeabilization of *P. aeruginosa* ATCC 9027 by these peptides. EtBr will only fluoresce if it is able to permeate both the inner and outer membranes through pores, and intercalate into the bacterial nucleic acid. The uptake of EtBr was significantly higher in the presence of DRGN-1 or LL-37 than in the presence of VK25 or the untreated control (Fig. 3a), suggesting significant disruption of the bacterial cytoplasmic membrane.

The effect of CAMPs on the membrane potential was measured by monitoring the membrane potential using the fluorescent dye 3,3'-dipropylthiadicarbocyanine iodide (DiSC_3_(5)) (Fig. 3b).^20^ DiSC_3_(5) dye is taken up by bacterial cell membranes in proportion to the electrical potential gradient across the bacterial cytoplasmic membrane. The dye concentrates in the cytoplasmic membrane, where it is able to self-quench its own fluorescence. Any compound that can permeabilize the cytoplasmic membrane and thus depolarize the gradient, such as an antimicrobial peptide, will lead to the release of DiSC_3_(5) and thus leads to an increase in fluorescence.^21^ The peptides were tested against *P. aeruginosa* ATCC 9027 at a concentration of 20 µg/ml, which was expected to cause a large and immediate increase in fluorescence (indicative of loss of membrane potential). DRGN-1 showed ~10% depolarization relative to Triton X-100 (which served as the “100%” positive control), while VK25 did not have any effect. These results suggest that in addition to membrane disruption, DRGN-1, much more than VK25, permeabilizes the membranes and slightly depolarizes the membrane potential.

### Structure analysis of DRGN-1 by circular dichroism (CD) spectroscopy

**Fig. 4:**
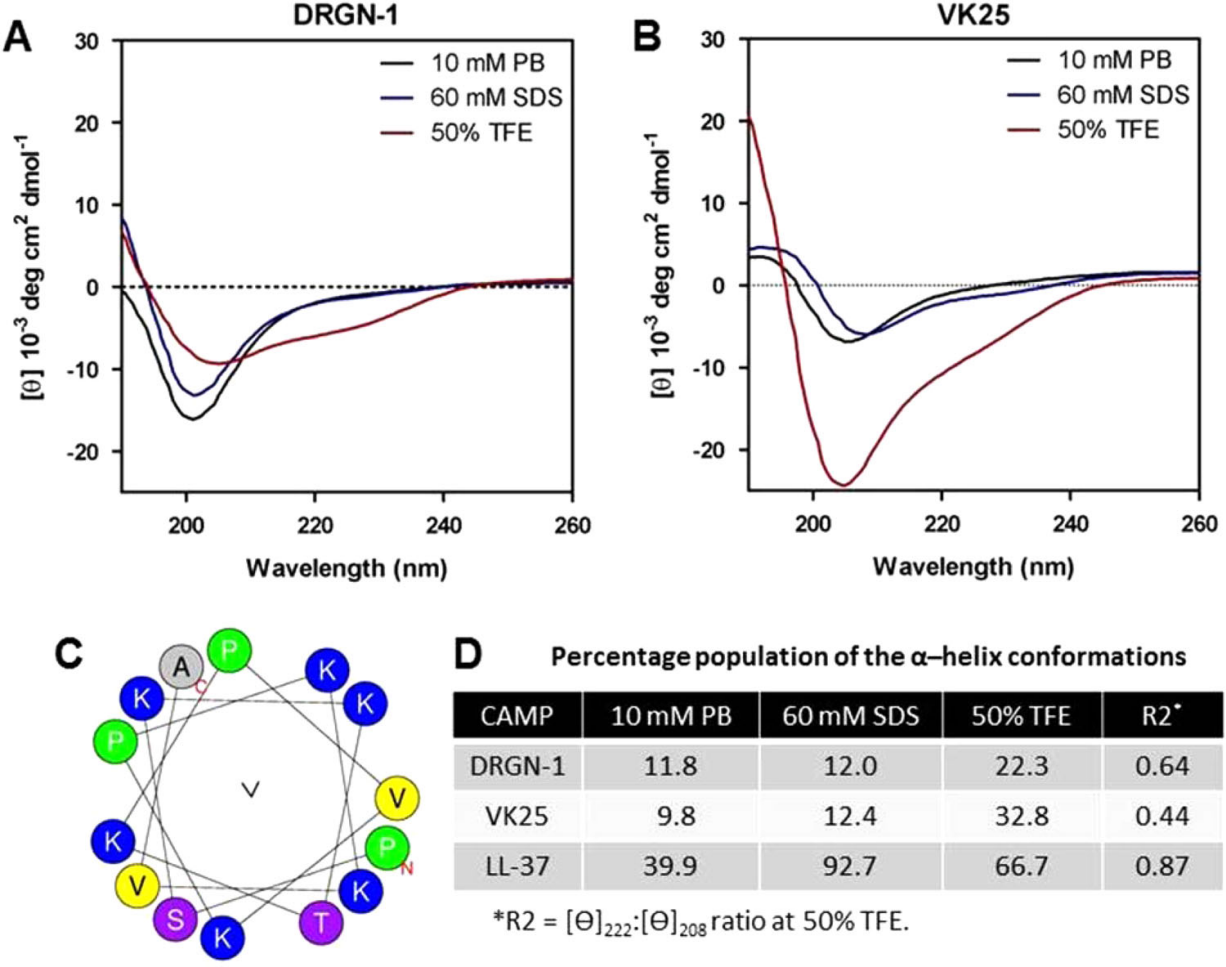
CD spectra of peptides in the presence of SDS or TFE. **a**, **b** CD spectra of 100 μg/ml concentration of peptides in the presence of the indicated concentrations of sodium phosphate buffer (PB), SDS or TFE. **c** Helical wheel plot of DRGN-1 predicted by HeliQuest. **d** Composition of *α*-helix conformation calculated from the ellipticity value at 222 nm using the relation (% *α*-helix = ([*θ*_222_]−3000)/(−36000−3000)). The ratio [*θ*]_222_/[*θ*]_208_ obtained by CD spectroscopy is defined as R2 value. R2 >1 is the hallmark of coiled-coil *α*-helical structure with >80% helical content (32)

The reversal of two N-terminal amino acids (ser–pro) in a histone H1-derived peptide of *V. komodoensis* caused drastic changes in antimicrobial activity, anti-biofilm activity, membrane permeability, and DNA binding. Therefore, we sought to determine the secondary structure of the peptides in the membrane-mimicking media, sodium dodecyl sulfate (SDS) and trifluoroethanol (TFE) (Fig. 4) using CD. Cathelicidin AMPs (such as LL-37) maintain a random or disordered structure until associated with a bacterial membrane or detergent micelle.^22^ The detergent SDS is able to form micelles that have a negatively charged surface,^23^ which mimics the bacterial membrane and forces the CAMP into a more ordered conformation such as a helix.^24, 25^ TFE is used in CD to promote a helical structure^26^ and stabilize secondary structure.^27^

By qualitatively evaluating the spectra, we conclude that DRGN-1 and VK25 showed a random coil conformation in aqueous solution (10 mM phosphate buffer) and 60 mM SDS solution. The *α*-helical content of DRGN-1 and VK25 were 22.3 and 32.8% in 50% TFE, respectively, with the percent contribution to secondary structure was measured using methods determined by Raussens *et al*.^28^ Both DRGN-1 and VK25 have a very low hydrophobic moment (0.013 and 0.088, respectively) compared with the highly *α*-helical LL-37 (0.521), suggesting that DRGN-1 and VK25 are not *α*-helical (Table 2). Nevertheless, DRGN-1 (0.64) displays a slightly larger R2 value, the hallmark of the coiled-coil *α*-helical structure in 50% TFE, than VK25 (0.44). It has been shown by several groups that TFE can induce and support *α*-helical structures in peptides.^29, 30^ These results suggest that the reversal of amino acids at the N-terminal contributes to an overall conformational change.

**Table 2:** Summary of CAMPs used in this study

CAMP	Sequence	Net charge	MW	Hydro-phobicity (H)^a^	Hydro-phobic moment (µH)^a^	AMP probability (SVM)^b^
DRGN-1	PSKKTKPVKPKKVA	+6	1535.95	−0.058	0.013	0.866
VK25	SPKKTKPVKPKKVA	+6	1535.95	−0.058	0.088	0.866
LL-37	LLGDFFRKSKEKIGKEFKRIVQRIKDFLRNLVPRTES	+6	4493.3	0.201	0.521	0.762

*SVM* support vector machine

^a^ Physicochemical parameter (hydrophobicity and hydrophobic moment) were calculated by HeliQuest, a web server to screen sequences with specific alpha-helical properties Gautier *et al*.^68^

^b^ AMP probability was predicted by a web-based prediction tool CAMP (http://www.camp.bicnirrh.res.in/predict/) using support vector machine SVM algorithm.

### Efficacy of DRGN-1 on in vivo wound healing

We have demonstrated that DRGN-1 peptide significantly inhibited biofilms of *P. aeruginosa* and *S. aureus* separately, and a mixed biofilm. Since biofilm may protect bacteria during infection, we sought to test whether application of DRGN-1 to an infected wound would promote clearance and healing.

To evaluate the impact of a mixed biofilm infection on an open wound, we developed a wound model using BALB/c mice. We have previously characterized *P. aeruginosa* and *S. aureus*-mixed biofilms for their heterogeneity and composition;^19^ these biofilms were generated and applied to a mouse wound. In our model, we will test whether wound healing in *P. aeruginosa* and *S. aureus* biofilm*-*infected skin wounds is significantly improved following peptide treatment compared to an untreated, infected wound, following the design of other published studies.^31^

**Fig. 5:**
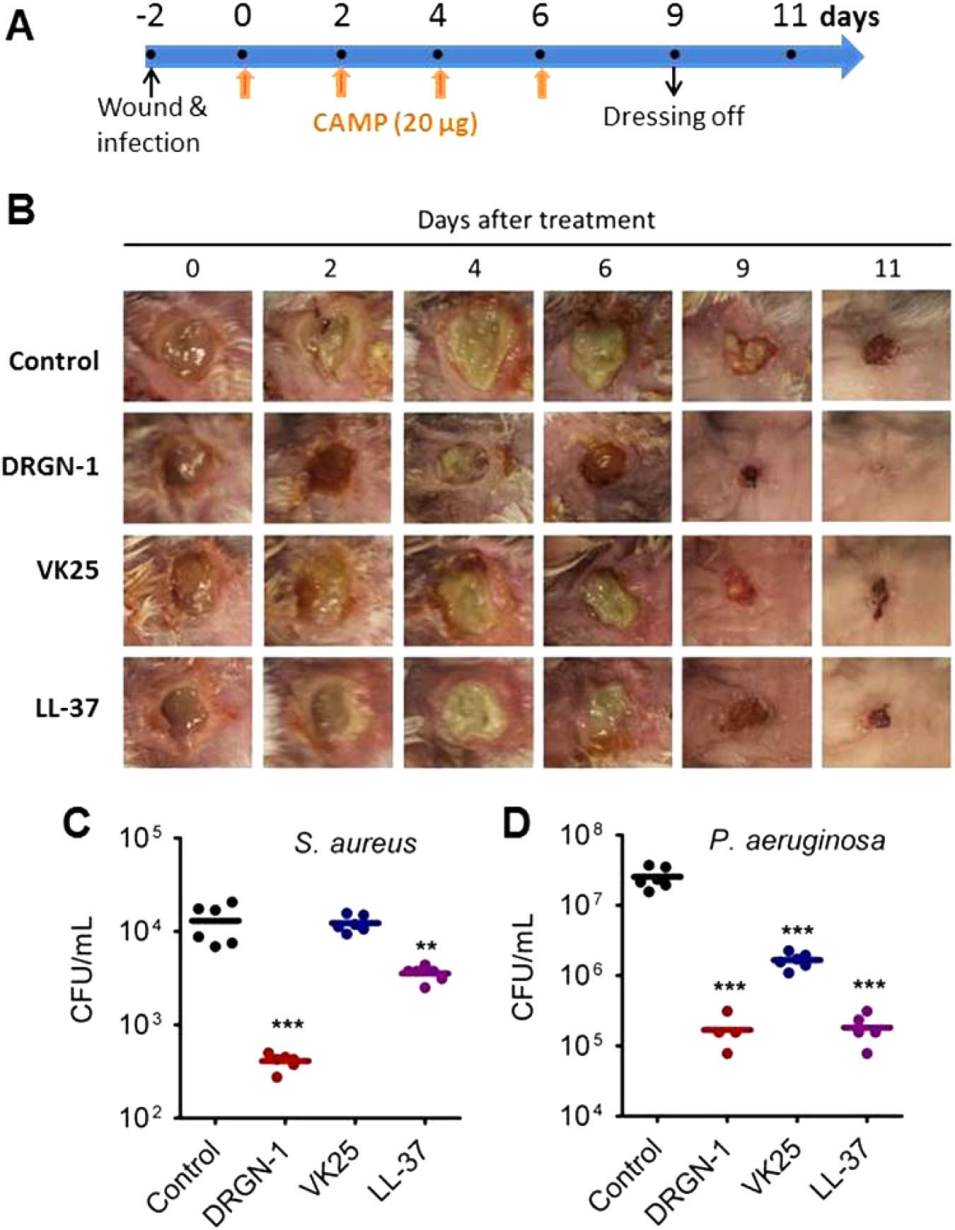
Efficacy in wound healing of peptides in a mixed biofilm-infected mouse skin wound model. **a** Schematic experimental design that includes the timing of infection, drug treatments, dressing off. **b** Effect of peptides on closure of *P. aeruginosa/ S. aureus*-infected wounds in mice. Wounds shown are representative of the group. Six-millimeter-diameter excisions were made on the back of mice. Each wound was infected with a mixed biofilm (*P. aeruginosa/ S. aureus*) grown on a polycarbonate membrane for 2 days. DRGN-1, VK25 or LL-37 (20 µg) was topically treated every 2 days in 1% hypromellose (*n* = 6 mice per group). Mice were lightly anesthetized with isofluorane and photographed on the indicated days following treatment. **c**, **d** Assessment of bacterial colonization in *P. aeruginosa*/ *S. aureus*-infected wounds. Wound tissue samples were harvested on day 6 after treatment, and the number of CFU/wound was counted in a selective medium for each species. The median value in each group is shown as a horizontal bar (*n* = 6; **p < 0.01 and ***P < 0.001)

We have previously characterized a mixed biofilm of *P. aeruginosa* and *S. aureus*,^19^ which is applied to the wound as the source of infection. Using this model, we evaluated the potential clinical application of DRGN-1 against *P. aeruginosa/S. aureus* biofilm-infected wounds (Fig. 5). Full-thickness round wounds of 6 mm in diameter were made between the shoulder blades of mice and overlaid with a mixed biofilm of *P. aeruginosa/S. aureus* grown on agar for 2 days.^19^ The kinetics of wound closure was evaluated by measuring the original wound area before and after treatment with peptides. DRGN-1, VK25, and LL-37 (20 μg/wound) were applied topically to their specified groups every other day until day 6. Reconstitution buffer was used as a control in this experiment. Treatment with DRGN-1 on a mixed biofilm-infected wound significantly reduced wound size compared to the controls as soon as day 4 post treatment. When the wound dressing was taken off on day 6, wound healing sped up in both treated and untreated conditions as observed at day 9.

Wounds treated with DRGN-1 were consistently smaller than wounds treated with phosphate buffered saline (PBS) buffer, VK25, or LL-37 (Fig. 5b, Fig. S3). The results showed that the difference in size was apparent by day 4 after the first treatment. By day 11, the wounds treated with DRGN-1 were completely healed, while the wounds treated with PBS, VK25, and LL-37 were not. After 6 days, the first treatment (three treatments), the bacterial counts of *S. aureus* and *P. aeruginosa* were significantly reduced in the DRGN-1-treated and LL-37-treated groups compared to the control group (Fig. 5c, d). In contrast, there was no significant reduction in *S. aureus* and only a slight reduction in *P. aeruginosa* in VK25-treated wound areas. These results indicate that DRGN-1 accelerates skin wound closure and healing, and reduction of bacterial counts in the wounds of *P. aeruginosa/ S. aureus* biofilm-infected mice. When the wound dressings were taken off on day 6, wound healing was accelerated in both treated and untreated conditions due to the air exposure, as observed at day 9 and 11 post treatment. In addition, hematoxylin–eosin staining of skin at day 11 clearly demonstrated that the skin layers were completely rehabilitated in the DRGN-1-treated wounds (Fig. S4).

**Fig. 6:**
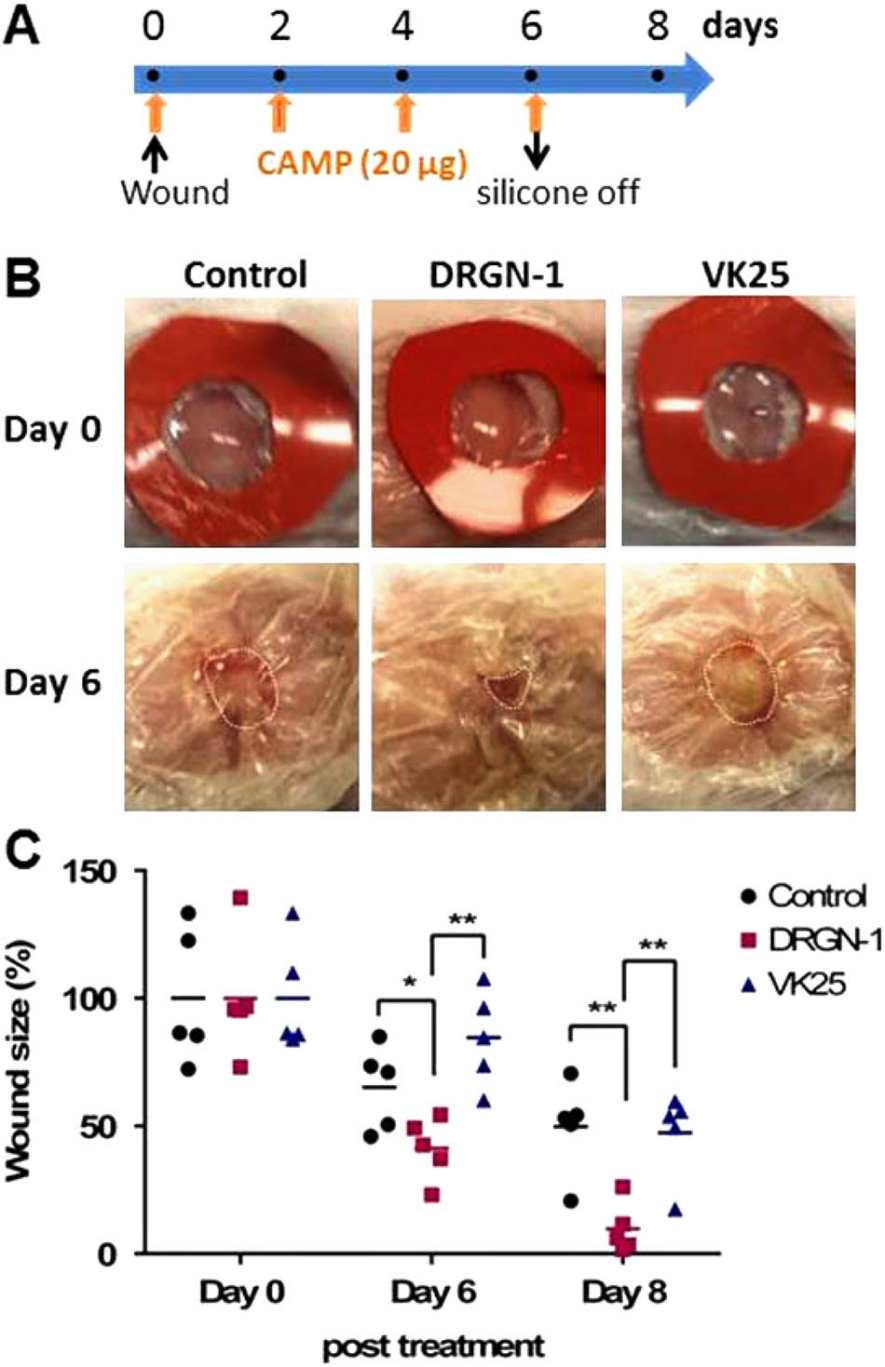
Wound healing of DRGN-1 in a mouse full-thickness skin (uninfected) wound model. **a** Schematic experimental design that includes the timing of drug treatments. **b** Effect of DRGN-1 on closure of full-thickness excisional wounds (6-mm-diameter) in mice. Each wound was treated with DRGN-1 or VK25 every 48 h (*n* = 5 mice per group). The same animal was lightly anesthetized and photographed on the indicated days following injury. **c** Changes in percentage of wound area at each time point in comparison to the original wound area. *P < 0.05 and **P < 0.01

To precisely dissect the effect of DRGN-1 on re-epithelialization and new tissue formation in wound, we then investigated the in vivo wound closure activity of DRGN-1 in a mouse full-thickness skin (uninfected) wound model. In this study, we used a silicone splint^32^ to avoid contraction of an extensive subcutaneous striated muscle layer, which is specific to mice, called the panniculus carnosus that is largely absent in humans.^33^ Full-thickness round wounds of 6 mm in diameter were made between the shoulder blades on mice, and the wound closure was evaluated by measurement of original wound area (%). When DRGN-1 or VK25 (20 μg/wound) was applied topically every 2 days post injury, DRGN-1-treated wounds were consistently smaller than wounds treated with PBS or VK25 (Fig. 6). At day 6, the wounds treated with DRGN-1 were smaller in area (41% compared to original wound area) than the control (65%) or VK25-treated (85%) wound. These results indicate that DRGN-1 peptide directly accelerates skin wound closure and healing in mice. In summary, wound healing was significantly enhanced by DRGN-1 in both uninfected and mixed biofilm (*P. aeruginosa* and *S. aureus*)-infected murine wounds.

### Efficacy of DRGN-1 on keratinocyte migration in a scratch-wound closure assay

Some CAMPs also have host-directed functions and thus CAMPs are also called host-defense peptides. One of the potential functions of CAMPs is the activation of keratinocyte migration resulting in the promotion of skin wound healing,^9, 34, 35^ which may contribute to the in vivo results we observed. Therefore, we tested the capacity of DRGN-1 to induce wound closure via this mechanism.

**Fig. 7:**
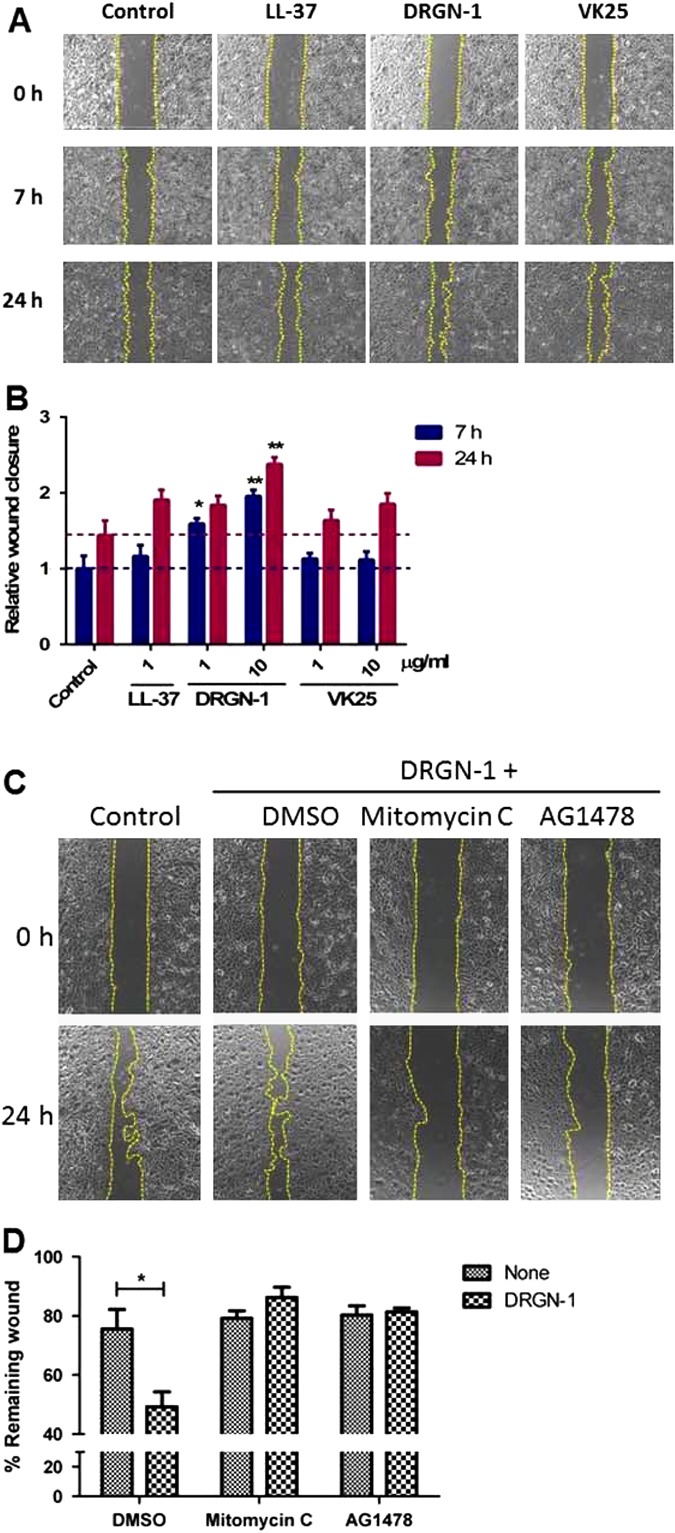
'Scratch' assay for HEKa cells. **a** Confluent cells in medium with HGKS were wounded by a scratch with a pipette tip, and ‘gap’ closure in the presence of peptides (10 µg/ml) was monitored by light microscopy at 0, 7 and 24 h post peptide treatment. **b** Relative wound closure of separate experiments using either 1 or 10 µg/ml of peptide as indicated was calculated from micrographs using ImageJ software. The results shown are combined from three independent experiments; error bars represent the mean ± SD. The *dotted lines* indicate the control values for easier comparison to the other bars. **c** HEKa cells were pre-incubated with 20 µM mitomycin C for 90 min or 0.2 µM AG1478 for 10 min and subsequently treated or not with 10 μg/ml DRGN-1, as indicated. In parallel, cells treated with the peptide alone were included for comparison. Cells incubated with medium served as a control (Ctrl). **d** Relative wound closure. All data are the means of at least three independent experiments ± SE. The levels of statistical significance between groups are indicated as follows: *P < 0.05

We first investigated whether CAMPs induced the migration of HEKa cells into a wound area using a scratch wound closure assay. The migration of cells into the wounded area was significantly increased in the presence of DRGN-1, as well as LL-37, compared to migration in the control within 7 and 24 h after wounding (Fig. 7a, b). There, significantly less wound closure activity was observed in the presence of VK25, which was slightly more than the control treatment (peptide reconstitution buffer). This suggests that part of the wound-healing benefit of DRGN-1 peptide is the promotion of keratinocyte migration. Subsequently, in order to determine whether the wound closure was influenced by an increased proliferation of keratinocytes upon their exposure to DRGN-1, the wound healing assay was carried out in the presence of 20 μM mitomycin C to block cell proliferation. As shown in Fig. 7c, d, mitomycin C strongly inhibited the migratory activity of keratinocytes induced by DRGN-1 (10 μg/ml). This suggests that the proliferation of HEKa cells highly contributes to the wound-healing effect produced by DRGN-1. To further understand the pathway by which DRGN-1 activates keratinocyte migration and proliferation, epidermal growth factor receptor (EGFR) was selected as a potentially activated kinase, because it has been reported to be central to the regulation of keratinocyte migration and proliferation.^35–37^ Previous studies have shown that LL-37 peptide can induce transactivation of the EGFR.^35, 38^

**Fig. 8:**
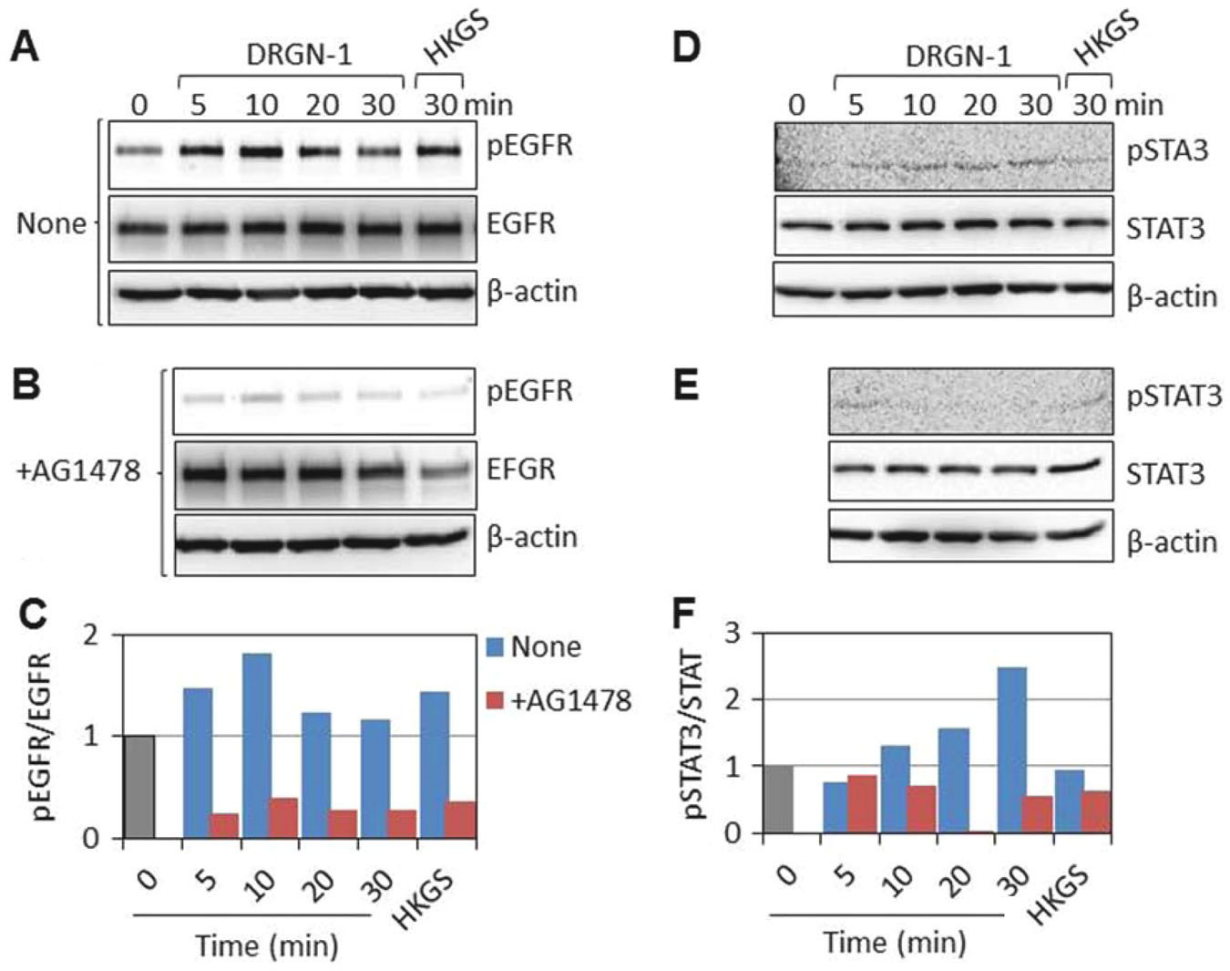
Activation of EGFR-STAT pathway by DRGN-1. **a**, **b**, **d**, **e** Subconfluent keratinocytes were starved for 2 h in HGKS-free medium and stimulated with 10 µg/ml DRGN-1. **b**, **e** For EGFR inhibition, cells were preincubated with 0.2 µM AG1478 for 10 min and subsequently treated with 10 μg/ml DRGN-1. The cells were harvested into lysis buffer at the indicated times. **a**, **b**, **d**, **e** The phosphorylation of EGFR (*p*-EGFR) and STAT3 (*p*-STAT3) was analyzed by Western blotting. EGFR indicates total EGFR protein. STAT3 indicates total STAT3 protein. **c**, **f** Densitometric analyses of the expression of phospho-protein relative to un-phosphorylated proteins from panels **a** & **b**, **d** & **e** above

AG1478 is an inhibitor of EGFR tyrosine kinase, which blocks the activation of EGFR.^38, 39^ Interestingly, pretreatment of HEKa cells with 0.2 μM AG1478 prevented cell migration induced by DRGN-1. To examine potential EGFR pathway activation by DRGN-1, we investigated EGFR phosphorylation by Western blotting with an anti-phospho-EGFR antibody. DRGN-1 treatment led to phosphorylation of the EGFR at 5 min and the phosphorylation persisted for 20 min, which was not observed in AG1478-treated cells (Fig. 8a–c). The amount of EGFR protein did not change during this time period and treatment with human keratinocyte growth supplements (HKGS) containing 0.2 ng/ml of human epidermal growth factor (EGF) was used as a positive control. EGFR signaling pathway initiates several signal transduction cascades, such as STAT3 (Signal Transducer and Activator of Transcription) and Akt with the functional consequence of enhanced cell migration.^40^ STAT3 was also phosphorylated 10–30 min after the addition of DRGN-1 (Fig. 8d–f). From these results, we conclude that DRGN-1 induces keratinocyte migration via EGFR-STAT3 pathway activation.

## Discussion

In this study, we evaluated the biological activities of the histone H1-derived peptide, VK25 identified from Komodo dragon blood and the N-terminally modified synthetic peptide DRGN-1. The peptide DRGN-1 has broad-spectrum activity, and is highly potent in antimicrobial and anti-biofilm assays. The bactericidal properties of a few histone-derived fragments (mainly H1 or H2B) have been recently reported in the literature.^41–45^ Some histone H1-derived CAMPs are found in various animals including fish^44, 46^ as well as humans.^47, 48^ We previously identified a potential histone H1-derived CAMP from Komodo dragons by a novel peptide-identification bioprospector platform.^13^ VK25, a peptide containing a native sequence from Komodo histone H1, did not display significant antimicrobial activity despite the predictive algorithm indicating a high probability of antimicrobial activity (Table 2). When the two N-terminal amino acids were switched from “SP” to “PS” in a new synthetic peptide that we named DRGN-1, it exhibited potent antimicrobial activity. Thus, we sought to investigate why this reversal induced the activity.

One possibility is that this switch may induce overall structural changes, thus enabling the peptides to exert differential activity.^5^ We determined the secondary structures of the peptides DRGN-1 and VK25 using CD spectroscopy. This data suggested that both peptides have a similar secondary structure, displaying typical random coil structure in aqueous solution. In the presence of 50% TFE, the peptides underwent the conformational changes to *α*-helical structure. However, the R2 ([*θ*]_222_:[*θ*]_208_ ratio) of DRGN-1 in 50% TFE was somewhat higher than VK25, suggesting a slightly more organized structure of DRGN-1 in this solution.^49^ When we examined the structure of CAMPs in a membrane-mimicking environment (SDS), there was no conformational difference between the peptides, supporting the idea that these CAMPs have a random coil structure in aqueous solutions. Although CD spectra suggest a slight conformational difference under TFE conditions, this may not be sufficient to explain the significantly different antimicrobial activity of these peptides.

Another potential explanation of VK25’s relative inactivity is peptide degradation by the “N-end rule” pathway, which is mediated by the molecular chaperones ClpXP or ClpS-ClpAP systems in bacteria. Our data suggested the hypothesis that the switching of the proline–serine sequence of the original VK25 peptide may contribute to DRGN-1 peptide’s antimicrobial activity, perhaps by the “N-end rule”. Notably, the DRGN-1 peptide displayed activities comparable to that of the “classic” AMP human cathelicidin LL-37 (EC_50_ = 0.24–2.7 µg/ml; 0.05–0.60 µM).^3, 4^ The “N-end rule” states that the half-life of a protein is determined by the nature of its N-terminal residue.^50^ Mogk *et al*. suggest that in prokaryotic cells, there is a “proteasome-like” machinery that includes an ATPase molecule (either ClpA, or ClpX) working with a proteolytic molecule such as Lon that is either covalently attached or such as ClpP, which is diffusible. This protein complex mediates the proteolysis of misfolded or specially tagged proteins in the bacterial cytosol.^50^ In particular, ClpXP system is able to recognize 5 motifs at the terminal regions: two located at the C terminus of proteins, and three N-terminal motifs.^51^ The N-terminal regions of Dps, *σ*^S^ and GlcB that start with serine (Ser) contain sequences that are necessary (and sufficient) to target those proteins for degradation by the ClpXP system.^51^ In this aspect, we speculate that since VK25 starts with Ser at the N-terminus, it may be a substrate for ClpXP. DRGN-1 starts with proline (pro) at the N-terminus and exhibits significant antimicrobial activity, suggesting it is resistant to ClpXP-mediated degradation. In addition, the addition of Pro at the N-terminus of the cell-permeable antimicrobial peptide Buforin II led to greater efficacy of this CAMP against *P. aeruginosa* and *S. aureus* (Chung and van Hoek, unpublished data). This implies alteration of CAMP degradation (thus improving the CAMP stability) by the “N-end rule” pathway. Further studies will be done to determine the contribution of the “N-end rule” to improve the activity of the DRGN-1 AMPs, to address remaining questions such as, the cytosolic location of ClpXP vs. the membrane-targeting activities of the AMPs.

To study the modes of antimicrobial action of DRGN-1 peptide, we first measured the intrinsic permeabilization of the bacterial membrane by the peptides with EtBr uptake and dysregulation of the membrane potential with DiSC_3_(5). EtBr uptake data indicated that DRGN-1, much more than VK25, moderately disrupts the cytoplasmic membrane causing leakage of intracellular components (although less than LL-37). In contrast, DRGN-1 induced a slow dissipation of the membrane potential when an environment-sensitive fluorescent probe DiSC_3_(5) was used. The results showed that DRGN-1 could induce bacterial membrane disruption, but does not drastically alter the proton motive force, potentially causing structural damage to the cytoplasmic membrane. Due to its random coil structure, DRGN-1 is likely to be a pore-forming and membrane-permeable peptide. Thus, it seems that the target sites of DRGN-1 are likely to be both the cell membrane itself and potentially the nucleic acids inside of the cell. A DNA binding assay (results not shown) supported this hypothesis, showing that DRGN-1 binds tightly to DNA, presumably resulting in inhibition of the macromolecular synthesis of the cell once inside the bacteria, similar to the mechanism of Buforin II^52^ and PR-39.^53^ Peptides such as DRGN-1 that have multiple modes of action against bacteria are of significant interest for potential future development, as it is less likely that bacteria will be able to generate resistance mechanisms against multiple modes of action.

DRGN-1 exhibited wound closure activity both in vitro and in vivo through the proposed mechanism of promotion of keratinocyte cell migration and proliferation. Thus, we have demonstrated that in addition to its antimicrobial activities, DRGN-1 has host-directed activities. DRGN-1 possesses potent, direct wound closure activity in an in vitro wound scratch assay using human keratinocytes. DRGN-1 elicited significantly stronger wound closure activity than VK25 by inducing more HEKa cell migration (similar to LL-37), accompanied by activation of EGFR. DRGN-1 treatment also induced phosphorylation of EGFR and STAT3 in HEKa cells. A recent study suggested that LL-37 can activate EGFR and lead to phosphorylation of STAT3, leading to keratinocyte migration^38^ through a mechanism of “transactivation”. That study demonstrated that LL-37 activates a metalloproteinase, which then cleaves the extracellular domain of heparin-binding EGF (HB-EGF). The soluble-form of HB-EGF then binds to and phosphorylates EGFR.^38^ Therefore, we postulate that DRGN-1 may act through EGFR, similar to the transactivation mechanism of LL-37, which is mediated by HB-EGF. We are continuing to determine the mechanism of DRGN-1 action on host cells.

In addition, topical application of DRGN-1 accelerated closure and healing of full-thickness excisional wounds in mice. Consistent with the in vitro scratch wound assay, there was no significant wound reduction in VK25-applied wound areas compared to the control wound. These results suggest that DRGN-1, significantly more than VK25, is active for wound closure in the in vivo wound environment, although the precise mechanistic differences between our two peptides towards the EGFR pathway has not yet been elucidated.

Anti-biofilm activity is another biological function exhibited by DRGN-1. Biofilms are structured multicellular communities of microorganisms that are associated with surfaces and resistant to antibiotic treatment.^54^ Our results showed that our short synthetic peptide DRGN-1 significantly inhibited biofilms of *P. aeruginosa* and *S. aureus* separately, as well as in mixed biofilms, similar to the human cathelicidin LL-37.^3, 4, 19^ Since the formation of biofilm protects bacteria during infection and allows for survival in a hostile environment,^55^ the inhibition of biofilm formation by DRGN-1 in a wound may serve as an additional mechanism to prevent bacterial survival in the host and promote healing.^6, 31^

To evaluate in the infected wounds the impact of DRGN-1 on a mixed biofilm infection of a wound, we developed a mouse model using BALB/c mice. We have previously characterized *P. aeruginosa* and *S. aureus*-mixed biofilms,^19^ which were applied to the wound as a source of infection. In our model, wound healing in *P. aeruginosa* and *S. aureus* biofilm*-*infected skin wounds was significantly delayed compared to an uninfected wound. Treatment with DRGN-1 on a mixed biofilm-infected wound significantly reduced wound size compared to the controls as soon as day 4 post treatment, and appeared to promote clearance of the biofilm from the wound. When the wound dressing was taken off on day 6, wound healing was faster in both treated and untreated conditions as observed at day 9. This observation supports a previously described hypothesis that oxygen is another essential component of wound healing.^56^ Thus, treatment with DRGN-1 significantly promoted the healing of mixed-biofilm-infected wounds in vivo.

One potential limitation of these experiments is that these studies are performed on the mouse wound model, as a first demonstration of in vivo activity prior to advancing towards clinical studies. Another potential limitation of this study is that our model of polymicrobial biofilm infection has two organisms, *P. aeruginosa* and *S. aureus*, not the full repertoire of all six ESKAPE pathogens that are known to potentially infect wounds. However, these studies suggest that DRGN-1 is a good candidate for additional studies and possible development as a topical therapeutic agent for infected wounds.

Taken together, our data on the antibacterial, anti-biofilm, and wound closure activities suggest that DRGN-1, a synthetic derivative of the histone H1-derived peptide VK25 from the Komodo dragon, might be a potent wound healing agent in polymicrobial infected wounds, with host-directed effects accomplished through the EGFR-STAT pathway as well as through anti-biofilm activity against the bacteria. Furthermore, DRGN-1 did not affect the viability of human keratinocytes and erythrocytes. These features suggest that DRGN-1 might be developed as a topical agent for infected wound treatment.

## Methods

### Bacterial strains and peptides

*Pseudomonas aeruginosa* ATCC 9027, *P. aeruginosa* PAO1 ATCC 15692, *Escherichia coli* O157:H7 ATCC 51659, and *Staphylococcus aureus* ATCC 25923 were obtained from the American Type Culture Collection (ATCC, Manassas, VA, USA). *F. novicida* U112 NR-13 was obtained from BEI Resources (Manassas, VA, USA). *P. aeruginosa* PAO1 cells that constitutively expressed GFP (PAO1 pTDK-GFP) and *S. aureus* SH1000 that expressed RFP (SH1000 pAH9-RFP) were kindly provided by Dr. Weibel^57^ and Dr. Boles,^58^ respectively. DRGN-1 and VK25 were synthesized by the conventional solid-phase method by ChinaPeptides (Suzhou, China). The crude compounds were purified to chromatographic homogeneity in the range of >95% by using reversed-phase high-performance liquid chromatography with a mass spectrometer by the company. LL-37 was purchased from AnaSpec (Fremont, CA, USA). All peptides were reconstituted in a buffer consisting of 20 mg bovine serum albumin and 10 µl acetic acid in 50 ml PBS.^59^ Table 2 summarizes characteristics of peptides used in this study.

### Assays for antimicrobial and anti-biofilm activity

*P. aeruginosa* and *S. aureus* were cultured in nutrient broth and mannitol salt broth, *E. coli* was cultured in Luria broth (LB), and *F. novicida* was cultured in tryptic soy broth containing 0.1% cysteine overnight at 37 °C. Overnight-cultured bacteria were numerated by a standard curve using optical density vs. colony forming units (CFU) per ml. The culture was resuspended in 10 mM sodium phosphate buffer (PB) (pH 7.4) and adjusted to a final amount of 2 × 10^6^ CFU/ml. For EC_50_ determination, peptides were used at variable concentrations (0.1–100 μg/ml) from a stock solution. Fifty microliters of each concentration of peptide solution was added to each corresponding well of a 96-well plate (BD Falcon, USA) and 50 μl of bacteria was added and incubated for 3 h at 37 °C. After dilution in the buffer, the bacteria were spotted on an agar plate of the corresponding media and colonies were numerated. Anti-biofilm activity was measured as previously described^19^ with the following modifications. For the mixed biofilm inhibition assays in the 96 well plate format, a 99:1 ratio of *S. aureus* and *P. aeruginosa* was used as the starting inoculation. Two-fold diluted peptides in TSB medium were incubated with bacteria (1:50 dilution in the same medium) overnight. A crystal violet assay was used for biofilm formation as described.^60^ A tube-based biofilm assay was performed as previously described.^61^

For confocal microscopy, bacteria (*P*. *aeruginosa* PAO1 pTDK-GFP and *S. aureus* SH1000 pAH9-RFP) were grown on the Lab-Tek II chamber slide (Thermo Scientific) for 24 h in TSB and incubated with the indicated amount of peptide. Biofilms attached to the glass were observed with a 40× objective using a Nikon TE2000-U confocal laser scanning microscope equipped with an argon ion laser.

### Cell infection

Adult human epidermal keratinocytes (HEKa) (Invitrogen) cells were seeded in 24-well plates (Corning) and grown until they reached confluence (48 h) in EpiLife medium supplemented with HKGS containing 0.2% v/v bovine pituitary extract, 5 µg/ml bovine insulin, 0.18 μg/ml hydrocortisone, 5 µg/ml bovine transferrin, and 0.2 ng/ml human EGF (Invitrogen). The bacterial strain *P. aeruginosa* ATCC 9027 was grown in brain heart infusion (BHI) media at 37 °C with mild shaking and then harvested by centrifugation. Bacterial suspension (MOI 10) in EpiLife medium was co-incubated with keratinocytes for 2 h at 37 °C and 5% CO_2_. The medium was aspirated and the cells were washed three times with PBS to remove non-adherent bacteria. In order to kill extracellular bacteria, cells were incubated for 1 h in EpiLife without HKGS supplemented with 100 μg/ml of gentamicin. Afterwards, the medium was aspirated and the infected cells were washed three times, as described above. Three hundred microliters of peptide solution (30 µg/ml) in EpiLife without HKGS was added to each well and the plate was incubated for 2 h at 37 °C and 5% CO_2_. Then, the peptide solution was removed and the cells were washed again with PBS three times and lysed with 100 μl of 0.1% Triton X-100 in PBS for 5 min at room temperature. The resulting bacterial suspension was sonicated in a water bath for 10 min, to break up possible clumps, and appropriate dilutions were plated on agar plates for colony counts; the CFU were counted after 24 h at 37 °C.

### Membrane permeability

For intrinsic permeabilization of bacterial membrane, EtBr uptake was in performed 10 mM potassium phosphate buffer (pH 7.2) as described^62^ with some modifications. Overnight cultures of *P. aeruginosa* ATCC 9027 in BHI were transferred to fresh medium and grown to OD_600_ values of 0.5–0.6 (log phase). Cells were harvested and resuspended in 10 mM potassium phosphate buffer (pH 7.2) at a final OD_600_ of 0.12–0.15. The cells (40 µl) were added to 50 μl of 12 µM EtBr, followed by the peptide (20 µg/ml final) samples after 30 s. Excitation and emission wavelengths were set at 545 and 600 nm, respectively. The increase in fluorescence as a result of partitioning of EtBr into the cytosol was measured 20 min after the addition of peptides using a fluorescence spectrophotometer (Tecan Safire^2^ Multi-detection Microplate Reader). Membrane potential was measured by DiSC_3_(5) as described.^63^ Briefly, mid-log phase bacteria were suspended in 5 mM HEPES (4-(2-hydroxyethyl)-1-piperazineethanesulfonic acid) buffer containing 20 mM glucose (OD = 0.05) and DiSC_3_(5) (1 µM final) was added for 1 h at room temperature. Then KCl was added at 0.1 M of final concentration. After addition with peptide (20 µg/ml) or TX-100 (final 0.1%), fluorescence was excited at 622 nm (bandwidth 5 nm) and monitored at 670 nm for emission (bandwidth 20 nm).

### Circular dichroism (CD) analysis

CD spectra of the peptides were collected using a Jasco J-1500 spectropolarimeter as previously described.^3^ Data was collected in a 0.1 cm path length cuvette, with a chamber temperature of 25 °C. The spectra were collected from 190 to 260 nm with 0.1-nm intervals. Three scans per sample were averaged. All peptides were analyzed at 100 μg/ml concentration in three different mediums: 10 mM PB (pH 7), 50% (v/v) TFE in 10 mM PB (pH 7), and 60 mM SDS in 10 mM PB (pH 7). The ellipticity at 222 nm is used as a means of providing a rough estimate of the degree of peptide helicity under the three conditions investigated (10 mM PB, 60 mM SDS, and 50% TFE) consistent with the work reported by Raussens, *et al*.^28^ and as previously reported by our group.^22^ We performed the CD scan across the wavelengths shown in Fig. 4. In 10 mM PB and 60 mM SDS conditions, the peptides appeared to show very low helical content, however the results in TFE suggest higher helical character. Percent contribution to secondary structure was measured using methods determined by Raussens *et al*.^28^ We have applied a second approach to estimating helicity for the peptides in TFE. We have used the ratios of the signals at 222 and 208 nm (R2 = [Θ]_222 nm_:[Θ]_208 nm_), as has been reported by Graddis, *et al*.^49^ and Juba, *et al*., to provide a second means of estimating potential for helical structure.

### In vitro scratch wound closure assays

In vitro wound closure was assayed in confluent cell monolayers of HEKa (Invitrogen) as described.^34^ Cells were seeded in 6-well plates (Corning, NY, USA) and grown until they reached confluence (48 h) in EpiLife medium supplemented with HKGS. Cells were starved for 18 h in EpiLife without HKGS and then scratched once vertically with a 200-μl pipette tip to create an artificial wound. For inhibitor studies, mitomycin C (20 µM final) and AG1478 (0.2 µM final) were added 90 and 10 min prior to scratch generation. After being washed twice with PBS to remove cellular debris, cells were treated with 10 µg (Fig. 7a) or 1 and 10 µg (Fig. 7b) DRGN-1, VK25 or LL-37. PBS was used as a negative control. Cells were photographed before peptide treatment and 7 and 24 h after peptide treatment using an EVOS^®^ Cell Imaging Systems (Invitrogen). The same image fields were captured and the wound areas were estimated by ImageJ software.^64^

### MTT assay

The toxic effect of the peptides on adult HEKa cells was evaluated using the MTT dye colorimetric method.^65^ MTT’s intensity of the color is directly proportional to the number of metabolically active cells. Keratinocytes were plated in triplicate wells of a 96-well plate, at 4 × 10^4^ cells/well in EpiLife medium supplemented with HKGS. After overnight incubation at 37 °C in a 5% CO_2_ atmosphere, the plate was incubated with the peptides at different concentrations for 2 h, and 0.5 mg/ml of MTT was added. After 4 h of incubation, the formazan crystals were dissolved by adding 100 μl of acidified isopropanol, and the absorbance of each well was measured at 570 nm using a microplate reader (PowerWave X, BioTek).

### Hemolytic assay

The hemolytic activities of peptides was determined using sheep erythrocytes^66^ in an assay adapted to a microtiter plate format. Briefly, erythrocytes were prepared by centrifuging fresh defibrinated blood, re-suspending the pelleted cells in sterile PBS. The cells were washed with PBS three times. In the final wash, the cells were resuspended in 0.75 ml PBS. A 2% erythrocyte suspension was prepared for the assay and incubated with the indicated amount of peptide.

### Western blotting

Semi-confluent HEKa cells were serum-starved for 3 h and then stimulated with 10 µg/ml DRGN-1 or HKGS for 5–30 min at 37 °C. AG1478 (0.2 µM final) was added 10 min prior to DRGN-1 or HKGS treatment. Cells were lysed with 2× Laemli sample buffer (Bio-Rad) containing 5 mM sodium fluoride and 2 mM sodium vanadate. Cell lysates were separated on a 4–12 % SDS-PAGE gel and transferred to polyvinylidene difluoride membrane using an iBlot system (Invitrogen). The membranes were probed with antibodies specific to phospho-EGFR and EGFR (Cell Signaling). The membranes were then treated with horseradish peroxidase-conjugated donkey anti-rabbit IgG (Abcam) and developed by SuperSignal West Femto Chemiluminescent Substrate (Fisher Scientific) under GelDoc Imaging System (Bio-Rad).

### Animal model of wound healing

Female BALB/c mice (12 weeks old) were purchased from Jackson Laboratory (Bar Harbor, ME, USA). The Institutional Animal Care and Use Committee at George Mason University and ACURO approved all protocols (Protocol No. 0271). The study adhered to the NIH Guide for the Care and Use of Laboratory Animals. The mice were anesthetized with isoflurane and the dorsal skin was shaved to remove hair. The next day, a superficial wound was created between the shoulder blades of the mice using a 6-mm biopsy punch. The wounds were topically treated with 20 μl of a gel containing 20 μg DRGN-1 or VK25 in 1% hypromellose (hydroxy propyl methyl cellulose, HPMC, Sigma) every 48 h (*n* = 5 animals per group) and covered with a Tegaderm-type semiocclusive dressing (3M, St. Paul, MN) attached to a silicone disc (0.5 mm thick, Life Technologies) by Mastisol liquid (Eloquest Healthcare) for study involving only peptides. Each wound site was digitally photographed at the indicated time intervals and wound areas were determined on photographs using ImageJ. Changes in the wound areas over time were expressed as the percentage of the original wound areas.

For infected wounds, *S. aureus* ATCC 25923 and *P. aeruginosa* ATCC 9027 were mixed at an initial ratio of 1000:1 and grown on a 6-mm polycarbonate filter on Nutrient agar plates overnight as previously described.^19^ The resulting biofilm was applied to the biopsy wound and covered with a Tegaderm dressing adhered to the skin with Mastisol liquid. Mice were housed individually with ad libitum rodent chow and water for 2 days. The wounds (5 mice per group) were topically treated with 20 μl containing 20 μg peptide with 1% hypromellose in 10 mM phosphate buffer every 2 days until 6 days from the first treatment.^67^ Each wound site was then digitally photographed at the indicated time intervals, and wound areas were determined on photographs using ImageJ software. Changes in wound areas over time were expressed as the percentage of the original wound areas. To assess bacterial colonization, wounds were harvested at day 6 post treatment and homogenized in PBS. Mannitol salt agar was used to selectively culture *S. aureus*, and tryptic soy agar with triclosan or centrimide agar was used to selectively culture *P. aeruginosa*.^19^ At day 11 post-treatment, all mice were sacrificed and tissue samples were harvested in a 4% paraformaldehyde solution. The sections of fixed skin were stained with hematoxylin and eosin for further histological analysis by AML Laboratories (Baltimore, MD, USA).

### Statistical analysis

*P*-values were calculated by one-way ANOVA or Student’s *t*-test. Statistical significance was set at *P* < 0.05. Data are presented as arithmetic mean ± SD. Each experiment was repeated at least three times independently.

## Electronic supplementary material

Supplementary Material

## Electronic supplementary material

Supplementary Information accompanies the paper on the *npj Biofilms and Microbiomes* website (doi:10.1038/s41522-017-0017-2).
