# Supplementary material for: Komodo dragon-inspired synthetic peptide DRGN-1 promotes wound-healing of a mixed-biofilm infected wound
Source: NPJ Biofilms Microbiomes. 2017 Apr 11;3:9. doi: 10.1038/s41522-017-0017-2 (PMC5445593; doi:10.1038/s41522-017-0017-2)
Supplement: Supplementary file 1 — Supplementary Material [file 41522_2017_17_MOESM1_ESM.docx]

**NPJBIOFILMS-00150R**

**Supplementary data**

**Fig. S1.** Schematic diagram outlining relevant experimental procedures which lead to the identification of novel antimicrobial peptides such as VK25 from Komodo dragon by Bioprospector capture and *de novo* peptide sequencing as described ^1^.

**Fig. S2.** Hemolytic activity of antimicrobial peptides. A fresh sheep erythrocyte suspension was incubated with each peptide. Release of hemoglobin into the supernatant was measured by absorbance at 567 nm to indicate membrane damage of erythrocytes.

**Fig. S3.** Changes in percentage of wound area of experiment shown in **Figure 5** at each time point in comparison to the original wound area. Wound area was determined from photographs using ImageJ. DRGN-1 vs PBS =P<0.05 on Day 4 and P<0.01 on Day 11. Error bars are shown representing the standard deviation of the measurements.

**Fig. S4.** Histologic analysis of *P. aeruginosa/S. aureus*-infected wounds. Sections from wound tissue samples harvested on day 11 after treatment were stained with hematoxylin and eosin. Black arrows indicate epidermal gaps, which determine the degree of wound closure. In panels, dermo-epidermal boundaries are outlined by dashed lines.


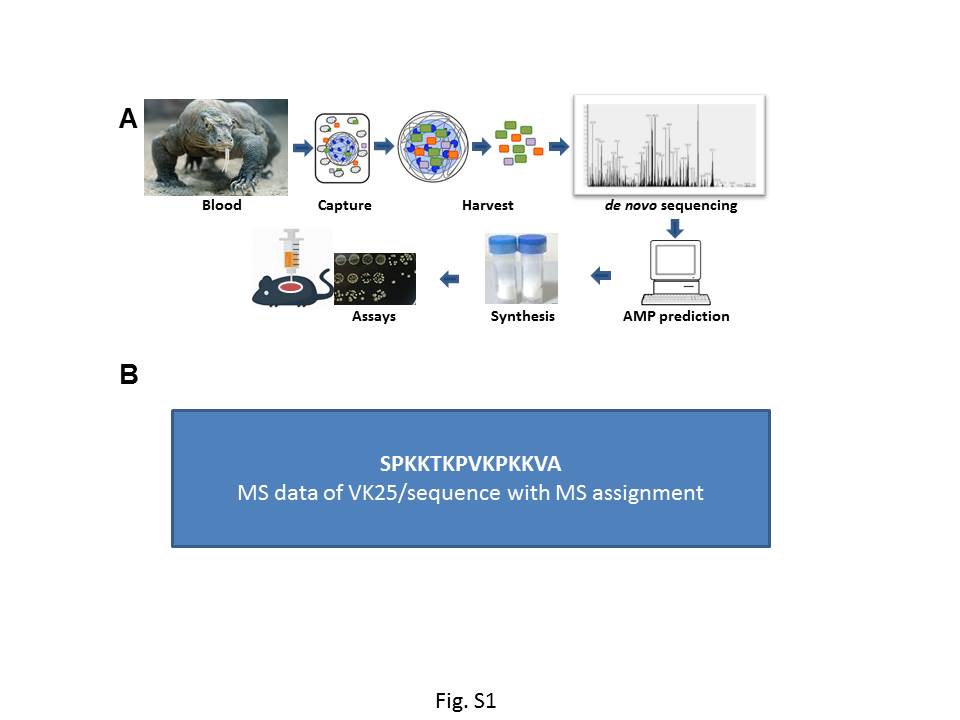


Fig S1


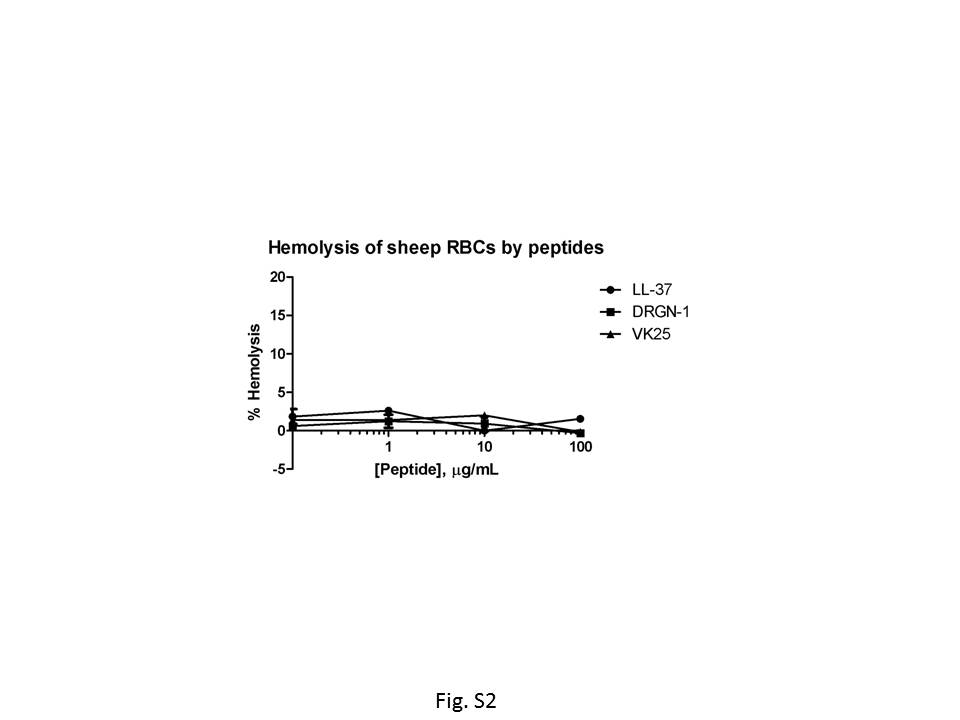


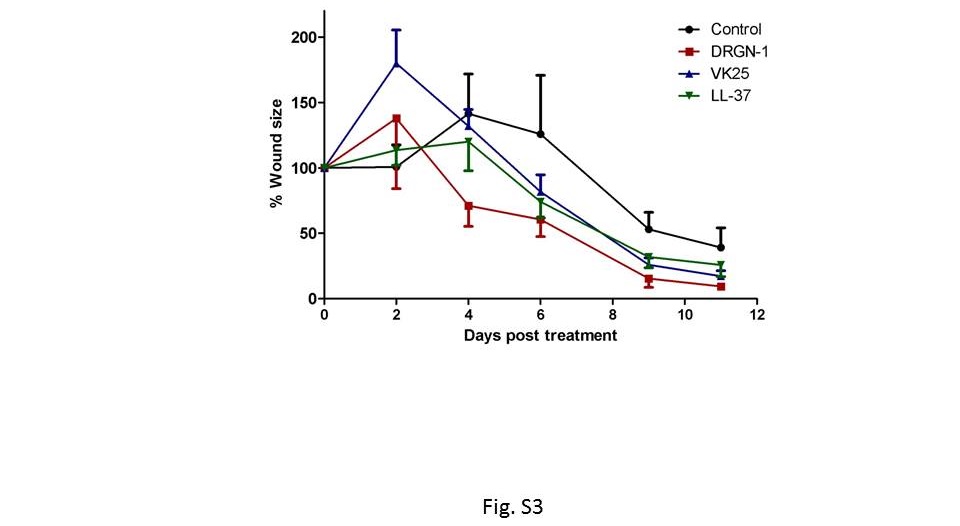


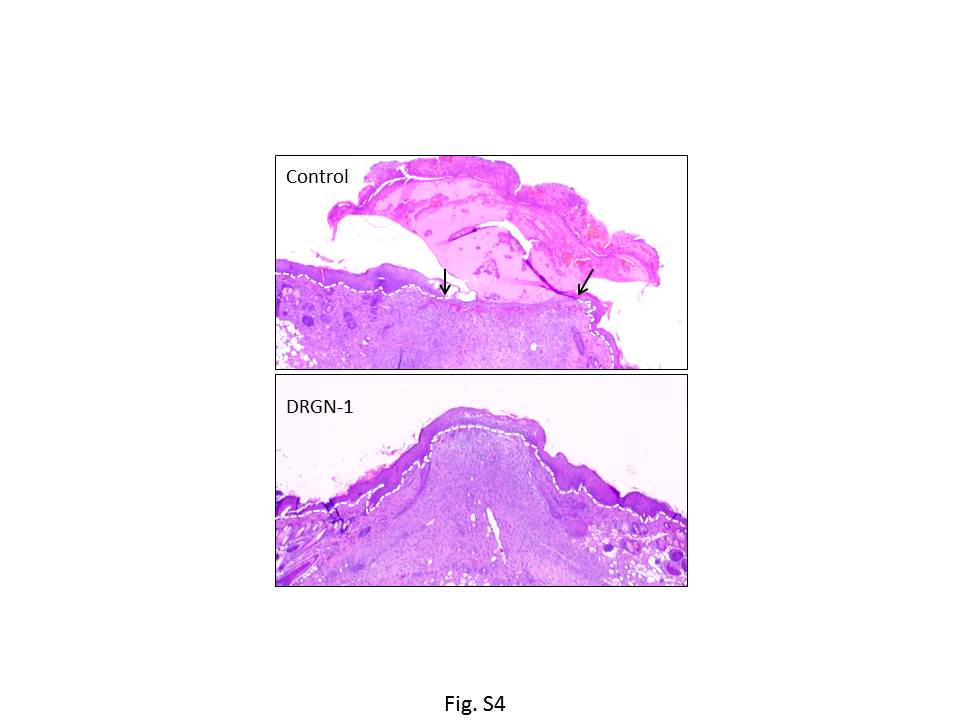


References and Notes:

1 Bishop, B. M. *et al.* Bioprospecting the American alligator (Alligator mississippiensis) host defense peptidome. *PloS one* **10**, e0117394, doi:10.1371/journal.pone.0117394 (2015).
